# Evaluating Alterations of the Oral Microbiome and Its Link to Oral Cancer among Betel Quid Chewers: Prospecting Reversal through Probiotic Intervention

**DOI:** 10.3390/pathogens12080996

**Published:** 2023-07-30

**Authors:** Prerna Diwan, Mohit Nirwan, Mayank Bahuguna, Shashi Prabha Kumari, James Wahlang, Rakesh Kumar Gupta

**Affiliations:** 1 Department of Microbiology, Ram Lal Anand College, University of Delhi, New Delhi 110021, India; mohitnirvan123@gmail.com (M.N.); mayankbahuguna21220@gmail.com (M.B.); shashiprabhakumari6@gmail.com (S.P.K.); rgupta1965@yahoo.com (R.K.G.); 2 Department of Biochemistry, St. Edmund’s College, Shillong 793003, India; wahlang.james@gmail.com

**Keywords:** microbiome, precancerous, lesions, betel quid, arecoline, areca nut

## Abstract

Areca nut and slaked lime, with or without tobacco wrapped in *Piper betle* leaf, prepared as betel quid, is extensively consumed as a masticatory product in many countries across the world. Betel Quid can promote the malignant transformation of oral lesions as well as trigger benign cellular and molecular changes. In the oral cavity, it causes changes at the compositional level in oral microbiota called dysbiosis. This dysbiosis may play an important role in Oral Cancer in betel quid chewers. The abnormal presence and increase of bacteria *Fusobacterium nucleatum*, *Capnocytophaga gingivalis*, *Prevotella melaninogenica*, *Peptostreptococcus* sp., *Porphyromonas gingivalis*, and *Streptococcus mitis* in saliva and/or other oral sites of the cancer patients has attracted frequent attention for its association with oral cancer development. In the present review, the authors have analysed the literature reports to revisit the oncogenic potential of betel quid and oral microbiome alterations, evaluating the potential of oral microbiota both as a driver and biomarker of oral cancer. The authors have also shared a perspective that the restoration of local microbiota can become a potentially therapeutic or prophylactic strategy for the delay or reversal of lip and oral cavity cancers, especially in high-risk population groups.

## 1. Introduction

**Figure 1:**
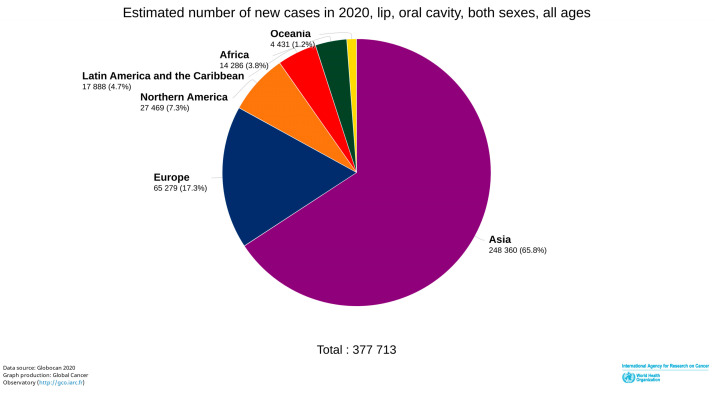
As per 2020 global data of International Agency for Research on Cancer and WHO, Asia has the highest number of the estimated new lip and oral cavity cancer cases registered in both sexes, all ages (http://gco.iarc.fr/; accessed on 24 December 2021).

**Figure 2:**
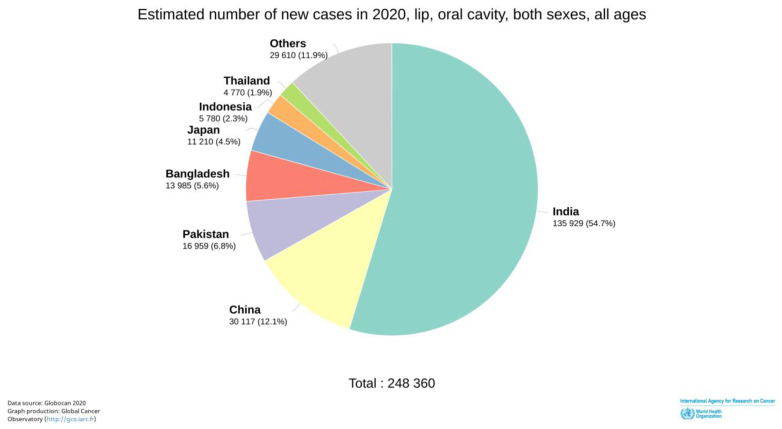
As per 2020 global data of International Agency for Research on Cancer and WHO, India has the highest number of the estimated new lip and oral cavity cancer cases registered in both sexes, all ages (http://gco.iarc.fr/; accessed on 24 December 2021).

Oral cancer (OC) is a major public health problem in South-Central Asia and Oceania (hotspots), with the highest estimated incidence rates in Papua New Guinea, Pakistan, Bangladesh, and India (one-third of total registered OC cases in 2020). According to the International Agency for Research on Cancer (IARC) Registries, GLOBOCAN (2020), Asia alone accounts for 65.8% of the estimated new cases of Lip and Oral cavity cancer (C00-C06) in comparison to Europe (65,279; 17.3%), North America (27,469; 7.3%), and the Caribbean region (17,888; 4.7%) (Figure 1) [1]. In Asia, India registered the highest number (36%) of C00-C06 in terms of total new cases in the South-East-Asia-specific region [1]. Following India, neighbouring countries Pakistan and Bangladesh report the highest incidences and mortality, thereby increasing the burden of OC in the South-East Asian belt (Figure 2) [1,2]. In this region, the age-old tradition of chewing betel quid (BQ) with or without tobacco is deeply rooted and believed to have an origin in moist tropical climates. The rampant chewing of BQ is due to its abundant availability at a cheap cost and the social and cultural imbibition of the practice.

BQ is prepared with betel leaf (*Piper betle*), areca nut (*Areca catechu*), catechu (*Acacia catechu*), and slaked lime with or without tobacco, popularly known as *Paan* in India and Pakistan. The Areca nut (AN) is mainly composed of polyphenols, alkaloids majorly including arecoline, and tannins [3]. Arecoline is a psychoactive agent that imparts cholinergic effects on the parasympathetic nervous system as an agonist for muscarinic acetylcholine receptors [3]. Therefore, the level of toxicity imparted by the alkaloid can be the primary cause of the formation of reactive oxygen species, reactive metabolic intermediates, nitrosamines, tissue inflammation, or other unknown toxic BQ components [4,5,6,7]. Further, an additional factor which plays a key role contributing to the toxicity is the duration of chewing BQ [8]. Therefore, BQ has been categorised as a Group 1 Carcinogen by International Agency for Research on Cancer [5]. A major carcinogen known as nitrosamine 3-methylnitrosaminopropionnitrile (MNPN), derived from AN, has been reported in the saliva of betel nut chewers and is also classified as a major carcinogen to humans (in Group 2B category) [9].

Long-term consumption of BQ causes the development of malignant lesions as well as oral sub-mucosal fibrosis (OSF), eventually leading to OC [10]. Various investigations have been conducted to comprehend and examine the relationship between the BQ chewing habit and histological changes including precancerous lesions that precede the progression of cancer [11,12]. BQ and tobacco chewers experience oral submucous fibrosis (OSMF), a precancerous condition characterised through inflammation and progressive fibrosis of submucosal oral tissues [13,14]. Recently, oral microbiota alterations have also emerged as a strong association with oral diseases, but this still needs further exploration [15,16,17]. In addition, BQ chewing may potentially affect the oral microorganisms due to their contents of various chemical and salivary alkalinity [18,19,20]. The oral microbiome is diverse in composition, which varies between individuals due to differences in their food habits, lifestyle, and health status. It evolves in stages from birth to adulthood [17]. An individual’s oral cavity harbours around 100–200 taxa [21]. A newborn displays the initial bacterial colonisation from the maternal parent source [17]. The metabolites produced from these initial colonisers facilitate the establishment of new microbes. Thereafter, oral microbiota complexity and diversity increases with age; for example, *Streptococcus* species are the first to colonise, and afterward, *Bacteroidetes* and *Spirochetes* become predominant in the oral cavity during the puberty stage [17]. The bacterial genera predominantly present in a healthy adult oral cavity include: *Prevotella*, *Capnocytophaga*, and *Bergeyella* (Bacteroidetes), *Neisseria*, *Cardiobacterium*, *Haemophilus*, *Campylobacter* (Proteobacteria), *Streptococcus*, *Granulicatella*, *Veillonella* (Firmicutes), *Fusobacterium* (Fusobacteria), *Rothia*, *Actinomyces*, *Corynebacterium*, and *Atopobium* (Actinobacteria) [22,23]. Among the various locales in the oral cavity, the tongue shows the highest diversity of bacteria and harbours *Veillonella atypical*, *Prevotella intermedia*, *P. gingivalis*, *Selenomonas* subspecies, *Actinobacillus actinomycetemcomitans*, and *Capnocytophaga* [24]. Over time, a ‘microbial homeostasis’ is established in the oral cavity, displaying a commensal and symbiotic relationship with the host, which provides different niches to establish, and, in return, oral microbes prevent the adhesion of non-commensal and pathogenic microbes [25]. However, when there is a change of microflora due to external interventions like BQ or tobacco chewing, a process called dysbiosis leads to the manifestation of oral diseases, including: dental caries, periodontitis, and OC. In dental caries, the enhanced growth of acidogenic and aciduric bacteria, including *Streptococcus mutans*, *Lactobacillus*, and *Bifidobacteria*, has been reported [26], and in case of periodontitis, members of the red complex viz. *Treponema denticola, Porphyromonas gingivalis*, and *Tannerella forsythia*, along with *Synergistetes*, *Saccharibacteria* (TM7), and *Scardovia wiggsiae* seem to dominate [27,28,29]. Additionally, there is currently mounting evidence linking the alterations in the oral microbiome with OC [30,31,32,33,34]. Despite these evidences, the role of the oral microbiome must be investigated further. In this review, an attempt has been made to first acquaint the reader about the effects of BQ and other substances (tobacco and alcohol) abused along with it, which are major risk factors associated with the onset of precancerous lesions and OC. We have further reviewed the link between OC and alterations in the oral microbiome and finally tried to gain insight into whether the reversal of oral microbiome dysbiosis in BQ and other substance abusers could potentially emerge as a viable prophylactic and/or therapeutic strategy for BQ-induced OC.

## 2. Betel Quid and Oral Cancer Prevalence

**Figure 3:**
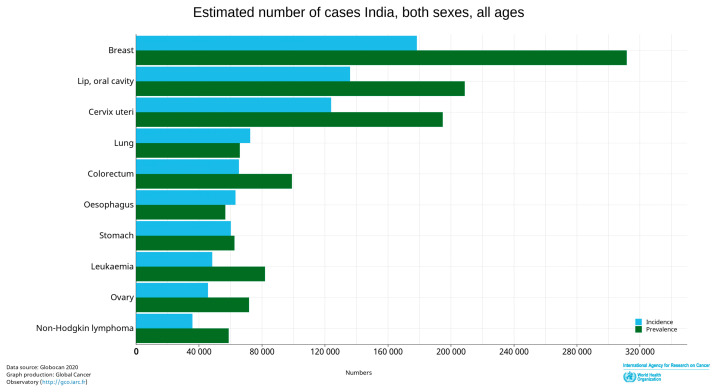
As per 2020 global data of the International Agency for Research on Cancer and WHO, in India, lip and oral cavity cancer cases newly registered in both sexes, all ages are second largest in number after breast cancer (http://gco.iarc.fr/; accessed on 24 December 2021).

According to the Globocan 2020 database on cancer (http://gco.iarc.fr/; accessed on 24 December 2021), globally, there are roughly 377,713 new cases of OC (C00-C06) with more than 130,590 deaths. The major burden of OC is borne by India, with the highest number of new cases of C00-C06 after breast cancer in 2020 (Figure 3). The statistics account for 135,929 new cases and 75,290 deaths. Cancer occurring in the lips, oral cavity, nasopharynx, and pharynx are included under the classification of oral cancer. The squamous epithelium, due to its superficial position, is firstly influenced by the carcinogens [35,36]. Ninety percent of OC cases globally are therefore oral squamous cell carcinoma (OSCC) [37]. Other habits, including tobacco, BQ, and alcohol consumption, are some of the major risk factors for OSCC [37].

AN, the seed of *Areca catechu* belonging to the family Palmaceae, is commonly and mistakenly called betel and is a constituent of BQ chewed along with *Piper betle* leaf [38]. AN is used in unripe, ripe, raw, baked, roasted, boiled, or fermented forms in BQ preparations. India stands as the largest AN producer (colloquially called *supari*) and consumer, partly due to its dependence [39]. Therefore, the onset of addiction occurs at an early age due to its easy availability in many attractive flavours and forms with or without tobacco at a low price. It is also popular among other Asian countries; however, the composition of BQ varies in different regions. For instance, in Taiwan, BQ is always taken without tobacco [40], unlike in India. It is chewed for its psycho-stimulating effects, to diminish hunger and constipation, to remove bad breath, and as a social practice. The medicinal benefits of BQ, including laxative, anti-ulcerative, anti-diarrhoeal, anti-helminthic, and anti-hypertension properties, are credited to its components, leading to a high consumption rate [41]. *Piper betle* leaf extracts have also been reported to have antibacterial activity against both Gram-negative and Gram-positive bacteria [42]. The alkaloid components of AN viz. arecoline, arecaidine, guvacoline, and guvacine are absorbed in the oral cavity because of ample time of contact as it is chewed gradually, leading to the onset of a number of physiological effects. While these effects are habit-associated and dose-dependent, they are more prominent in fresh and occasional chewers in comparison to habitual chewers, indicating BQ tolerance in them [43].

WHO also reported tobacco and alcohol as the two most important associated factors accounting for 75–90% of OC in people with the habit of chewing BQ and AN, and both are labelled as Group I carcinogens. A meta-analysis of earlier reported studies from South-East Asia displayed an increase in OC through smoking–drinking–BQ chewing interaction by 23–34 times, accounting for two-thirds of the cases in this region [44]. Table 1 captures studies connecting chewing and other substances with the rise in the risk of oral, pharyngeal, and oesophageal cancer in Asian countries as a prevalent habit. The following section reviews the studies elucidating the various underlying mechanisms for oral carcinogenesis due to BQ components [45].

## 3. Multifaceted Effects of Betel Quid Components in Oral Carcinogenesis

Arecoline and other alkaloids of AN in BQ lead to genotoxic and interfering effects in the DNA repair mechanism in the chewing population [64,65,66,67,68]. Studies have reported an accumulation of damaged mitochondrial DNA primarily in the form of deletions in BQ chewers [69]. Salivary arecoline persists in the oral cavity of the user for a long time after exposure to the AN and greatly enhances the adverse effects of the alkaloid [70]. Thus, a bridging concept could be established with BQ chewing and oral potential malignancy disorder (OPMD), OSF, and leukoplakia, ultimately leading to OC at the same site [59]. Oxidation of Arecoline in AN produces Arecoline N-oxide, which is an initiator of OC. It may be detoxified by N-acetylcysteine and block the cascade of cytotoxicity [9]. Cysteine found in human saliva converts to N-acetylcysteine and conjugates with arecoline N-oxide to form non-toxic arecoline N-oxide mercapturic acid. Arecoline has also been reported to up-regulate cystatin C in buccal mucosa fibroblasts, inducing a misbalance in extracellular matrix (ECM) synthesis and degradation by overexpression of cystatin C, which leads to OSF [71]. The presence of lime in BQ further greatly facilitates the penetration of arecoline in the mucosal barrier due to an increase in the salivary pH [70]. Arecoline metabolism to arecoline N-oxide by human-flavin-containing monooxygenase-3 generates a high level of Reactive Oxygen Species (ROS) in human saliva [72,73], which may induce cytogenetic damage observed in the oral cavity of BQ chewers [74]. BQ chewing with AN may also lead to exhaustion of the GSH (glutathione) oral levels, which is otherwise known to prevent damage to cellular components caused by ROS, exhibiting ROS-induced genotoxicity [75]. Thus, the presence of lime in BQ and ROS may have a role in the development of OC [76]. Arecoline N-oxide subsequently influences molecular pathways involving P53, NOTCH1, FAT1, and caspase-8 [77,78]. The role of iron and copper in the oral epithelium has also been suggested to be involved in the formation of ROS and, hence, leading to cancer progression. The presence of highly reactive oxygen species in the tumour sites with free iron is capable of generating H_2_O_2_ (hydrogen peroxide), which damages DNA, paving the way for cancer induction and progression [79]. Copper present in the AN can also magnify collagen formation and/or interfere with p53 signalling [80]. Therefore, compounds that can sequester the free iron and copper in the oral cavity have potential therapeutic value.

## 4. The Human Oral Microbiome, Dysbiosis, and Oral Cancer Development

**Figure 4:**
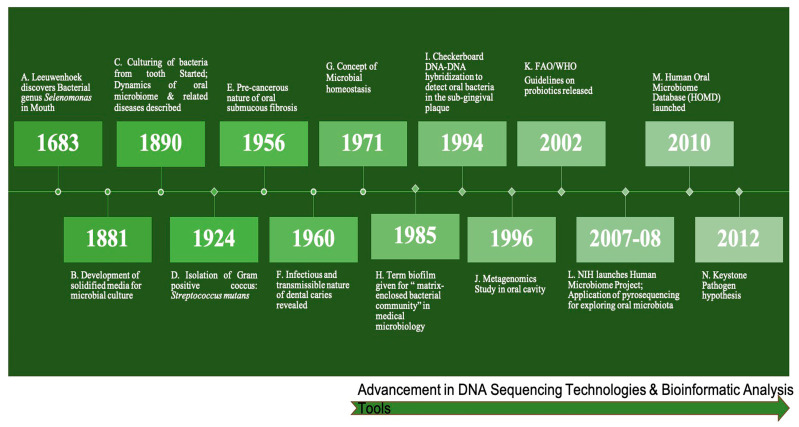
Timeline showing important milestones in Human Oral Microbiome research (A: [82]; B: [83] C: [84] Ring, 2002; D: [85]; E: [86]; F: [87]; G: [88]; H: [89]; I: [27]; J: [90] K: [91]; L: [92,93]; M: [94]; N: [95].

The human oral cavity extends from the vermilion border of the lip to the junction of the hard and soft palate in the mouth roof and circumvallate papillae on the tongue. Therefore, the oral cavity comprises the lips, commissures, tongue, mouth-floor, gingivae, buccal mucosa, retromolar trigone, and hard palate. The principal surface structure of the skin, lips, and mucous membranes of the oral cavity are called the squamous epithelium. The oral cavity maintains a temperature of 37 °C and pH of 6.5–7.5, conducive for oral bacteria to flourish. Since the first discovery of microbes in the human oral cavity by Leeuwenhoek (1683) and further progression of scientific studies revealing the oral microbiota secrets, we have come a long way ahead (Figure 4) [81]. After human gut microbiota, the human oral microbiota is the most varied and dynamic in composition. The oral microbiota has co-evolved with us, acquiring beneficial and deleterious properties. The oral microbiota consists of bacteria, viruses, fungi, protozoa, and archaebacteria. Among these, bacteria are the most abundant, with more than 700 species [81].

The Human Microbiome project led to the development of the Human Oral Microbiome Database (HOMD) in 2010 [96], and the current Expanded Human Oral Microbiome Database (eHOMD) includes a total of 771 microbial species [97], providing exhaustive literature on the bacterial species in the human aerodigestive tract (ADT), spanning the oro-nasopharyngeal cavity, sinuses, and oesophagus. The oral microbiome composition is variable in saliva and other parts of the oral cavity [98]. Most human oral bacteriome studies have characterised the culturable and nonculturable bacteria in the oral cavity under different conditions using around 1500 bp long 16S rRNA gene-based next-generation sequencing (NGS) technologies [99]. The 16S rRNA gene ic composed of nine hypervariable regions from V1 to V9. V1, V2, V3, and V4 sequences are extremely variable, and V5 exhibits the least variability. The PCR amplification of the V3 to V4 region generates a 500 bp long amplicon suitable for analysing bacterial diversity in oral cavity through metagenomic data analysis tools [100]. Several investigations have identified the oral cavity of healthy subjects being composed of a plethora of microbes that can be broadly categorised into six phyla: Firmicutes, Bacteroidetes, Proteobacteria, Actinobacteria, Spirochaetes, and Fusobacteria, and contain 96% of the taxa [101]. The remaining seven phyla, Euryarchaeota, Chlamydia, Chloroflexi, SR1, Synergistetes, Tenericutes, and TM7, constitute only 4% of the taxa [94].

The oral microbiota has been determined to be influenced by several factors, including light, atmospheric pressure, and redox potential, as well as nutrient sources, macro and micronutrients present in the saliva [102,103]. For example, the relative abundance of Betaproteobacteria and Fusobacteria is positively correlated with saturated fatty acids intake. Similarly, higher intake of sugars and water-soluble vitamins were found to be positively correlated with Lactobacillaceae and Fusobacteria, respectively [102]. Recently, differences in oral microbiota of the Indian population versus Western counterparts have also been reported, with the saliva of Indians harbouring more of Enterobacteriaceae, Proteobacteria, and Streptococcus. Among the Indian population itself, there are differences in microbiome composition in different geographical regions; for instance, *Chromobacterium* subspecies of bacteria were found to be enriched in the saliva of the Assamese population of the Indian subcontinent [24]. Such differences could be attributed to both diet and genetics of the population. Welch et al. (2019) highlighted a “site-specialist hypothesis”, stating that most microorganisms inhabiting the mouth are site specialists, mainly localised in one preferred habitat inside the mouth [104]. The same bacteria outside of its preferred site is typically found in much lower abundance, and the authors proposed a micro-scale analysis to understand how this site-specificity is achieved [104]. In a study by Caselli et al. (2020) of the oral microbiome of twenty healthy subjects, Streptococci was reported to be the most abundant bacteria in mucosal tissues (44–66% in the hard palate, oral mucosa, and keratinised gingiva), and 12–23% of the total genera was prevalent in the other sites (tongue, supragingival and subgingival plaque, saliva, and oral rinse) [98]. *Streptococcus mitis* is the most prevalent species, followed by *S. oralis*, *S. salivarius*, and *S. sanguinis*. *Neisseria*, *Prevotella*, *Rothia*, and *Haemophilus* were prominently present in most oral sites (representing 4–29% of the total bacteria). Anaerobes (*Actinomyces*, *Veillonella*, *Fusobacterium*) were particularly detectable in subgingival plaques, and *Simonsiella* was only found in the hard palate. In this study, the authors also reported the presence of a large number of strains, with genes conferring resistance functionality to different classes of antibiotics, macrolides, lincosamides, streptogramin, tetracycline, and quinolones [98].

A complex state of equilibrium existing in the primitive niche of the oral cavity represents a healthy state [105,106]. Changes in microbial species or taxa level diversity, due to environmental factors such as infection, diet, or lifestyle, cause a change in equilibrium leading to dysbiosis. This dysbiosis has emerged in many diseases in recent years and is a current subject of interest [107,108]. According to the ‘Keystone Pathogen Hypothesis’, certain microbial pathogens in low abundance are responsible for inflammatory diseases by bringing normal microbiota to a dysbiotic state [95]. Though many mechanisms have emerged with regard to the role of oral bacteria in cancer development, as reported in studies and reviewed by Zhang et al. (2018) [109], the question remains whether the oral microbiome is the cause or consequence of cancer. If the former is true, then does the microbiome have a role in the initiation or facilitation of the progress of cancer, and is there a microbial signature specific for OC which can be exploited as a biomarker? Some studies have tried to address these questions pertaining to the connection between bacteria and OC, investigating the possibility of a particular bacteria as a risk factor for OC. The role of the oral microbiome in OSCC through direct metabolism of carcinogens and inflammatory effects with higher representation of *C. gingivalis*, *P. melaninogenica*, and *S. mitis* in the saliva of healthy individuals has been reported [110]. *S. gordonii*, *S. mitis*, *S. oralis*, *S. salivarius*, *S. sanguinis*, and *Candida* [69] have been determined to facilitate an alcoholic metabolising mechanism which leads to the production of acetaldehyde supporting neoplastic transformations [111]. Nagy et al. (1998) reported significantly higher levels of *Porphyromonas* sp., and *Fusobacterium* sp. on OSCC tissue compared to the healthy mucosa region [112]. *F. nucleatum*, a Gram-negative non-spore-forming anaerobic prominently inflammatory *bacilli* with basins in the oral cavity and gastrointestinal tract, draws attention as an opportunistic pathogen because it has been isolated in a variety of infections and colorectal cancer biopsies [112,113]. *F. nucleatum* induces cytokines like tumor necrosis factor, interleukins-6, 8, 10, and 12, along with reactive oxygen species (ROS) and kinase generation, which helps in cancer progression [114]. It also acts as a bridging organism during the formation of biofilm in the oral cavity [115]. Species of Fusobacteria are hyperactive metabolically in the OSCC sites [79]. Oral leukoplakia, a precancerous condition, has also been shown to have an increased abundance of Fusobacteria and reduced levels of Firmicutes when compared to healthy controls [116,117,118,119]. However, contradicting findings lacking convincing evidence for the association of Fusobacteria with OC have also been reported [109,120], which may also be attributable to methodological differences between studies with respect to sampling sites and the selection of controls. Alterations in the NADPH oxidase activity and nitric oxide synthase (NOS) resulting in ROS and RNS accumulation leading to initial chronic inflammations and cancer development has been observed [121]. These effects are supported through the per-oxygenic oral bacterial classes such as *Bifidobacterium adolescentis*, *Lactobacillus acidophilus*, *L. fermentum*, *L. jensenii*, *L. minutus* [111], and other bacteria producing H_2_O_2_, which damages the macromolecules, DNA, proteins, and lipids [122]. *Bacteroides* and *Firmicutes* species also produce oncogenic substances, including sulfides and nitrosamines, in addition to fermenting the host’s excessive proteins [123].

Thus, oral microbiome alterations have been found to be associated with OC development. In addition, oral microbiome composition changes have also been shown to occur due to a number of lifestyle factors, including food intake (high sugar diet, etc.), hygiene, and betel nut, tobacco, and alcohol consumption [124,125]. With regard to this association in long-term BQ chewers, changes have been observed in the composition of normal oral microbiota as compared to non-chewers [124]. Interestingly, Hernandez et al. (2017) determined the alpha diversity (diversity within-sample) to be significantly lower among long-term (³10 years) betel nut chewers as compared to non-chewers [124]. Further, alpha diversity has also been shown to be significantly lower in chewers with oral lesions as compared to chewers and non-chewers with no lesions [124]. Among the oral microbial population, *Streptococcus infantis* consistently increased in BQ chewers as compared to past/non-chewers [126]. BQ chewers have also shown a higher abundance of *S. infantis* and decreased levels of *Parascardovia* and *Streptococccus* [124]. Another study reported a decrease in microbial diversity and richness in saliva following BQ chewing [127]. However, the study lacked microbiome analysis, so species-wise changes in the oral microbiota were not well understood. Table 2 and Table 3 summarise some more studies on oral microbiome changes associated with OCs.

Thus, dysbiosis in long-term exposure to substances like betel nut, tobacco, and alcohol has been shown to be associated with OSCC. Species of *Streptococcus*, *Prevotella*, *Rothia*, *Veillonella*, *Porphyromonas*, *Gemella*, *Peptostreptococcus*, *Porphyromonas*, *Micromonas*, *Dialister*, *Tanerella*, *Exiguobacterium oxidotolerans*, *Staphylococcus aureus*, *Eubacterium saburreum*, and *Capnocytophaga* were found to be elevated in oral and oesophageal cancer tissues as reported by previous studies [24,110]. Hooper et al. (2007) also observed significant differences between the microbial composition of tumorous and non-tumorous tissues [141]. In general, the cancer site’s microbiota was composed of saccharolytic and aciduric species [141]. The association of these bacteria with OSCC can be explained by inflammation-induced genotoxicity in epithelial cells caused by endotoxins [142]. *Prevotella melaninogenica*, *P.intermedia*, *P. nigrescens*, and *P. veroralis* have been frequently associated with OC patients [143]. The presence of other bacteria in the saliva of OC patients includes *Capnocytophaga gingivalis*, *Peptostreptococcus* sp., *Porphyromonas gingivalis*, and *Streptococcus mitis* [25]. *Porphyromonas gingivalis* is a keystone pathogen in adult periodontitis. These bacteria have also been suggested as diagnostic oncogenic markers [108,110]. A study by Zhang et al., 2020 [32] has shown that *Peptostreptococcaceae incertae sedis*, which occupied 0.04% in the normal oral buccal mucosa, escalated to 0.36% in OSCC sites and therefore might be a “keystone” pathogen with a critical role in carcinogenesis [32]. In earlier studies by Pushalkar et al., 2011; 2012 [144,145] *Streptococcus* and *Rothia* were found to be significantly lower as opposed to *Fusobacterium* in the cancer samples in comparison to normal and control samples [144,145]. Phylum Bacteroidetes was reported in higher abundance in OSCC patient’s cancerous and as well as normal tissues in comparison to pre-cancer and healthy control subjects [35]. Hence, this may serve as a biomarker of OSCC diagnosis. *P. gingivalis*, an intracellular bacterium, is an oral cavity coloniser [25]. In addition to causing periodontal destruction [25], it is potentially oncogenic by inhibition of mitochondrial apoptosis in the epithelial cells, which is regulated by the activation of Jak1/Akt/Stat3 signalling [113]. Patients with high levels of plasma antibodies against *P. gingivalis* have been shown to be at high risk of developing pancreatic and oesophageal cancer [146]. Elevated levels of *F. nucleatum* and *P. gingivalis* can be considered an indicator of tissue malignancy promoting cellular invasion and migration in OCs [25]. Zhou et al. (2020) proposed consortia of 12 bacteria, including *Streptococcus*, *Microbacterium*, *Fusobacterium*, *Brevundimonas*, and *Rhizobium*, with potential usage as biomarkers for predicting the risk of OSCC [147]. Unlike bacteria, there is very little evidence to associate fungal infections with OC [113].

## 5. Approach for the Reversal of Oral Microbiome Dysbiosis

The dysbiosis of the gut, skin, oral, and vaginal microbiome has been linked to many diseases and excellently reviewed [24,148,149,150,151,152,153,154]. Changes in microbial communities of the abovementioned human ecosystems and their application of probiotics—the living microbial strains—as an addition to diet, have gained interest in recent years [155,156,157,158,159]. Probiotics confer promising oral health benefits by restoring the balance of the oral microbiota [160]. Therefore, the oral cavity may be a potential target for probiotic interventions [161]. However, there are limited studies on the effects of probiotics on oral microbiome dysbiosis. *Bifidobacterium*, *Lactobacillus*, and *Streptococcus* strains are the most studied and qualified probiotic candidates [162,163]. In one of the most prevalent chronic oral diseases, dental caries, in both children and adults, the composition, structure, and function of oral microbial communities have been found to change [109] with microbial imbalance in terms of the dominance of acidogenic and acid-tolerant Gram-positive bacteria. *Lactobacillus*, *Bifidobacterium*, and *Streptococcus* have been reported to be effective in preventing dental caries [162]. Furthermore, diseases like periodontitis are one of the key oral diseases leading to OC, and gingivitis and periodontic infections explain the definite ability of certain probiotic lactobacilli as an antagonistic aid to the active pathogenic bacteria such as *Porphyromonas gingivitis* and *Aggregatibacter species* [164,165]. Ahuja and Ahuja (2021) reviewed the randomised controlled trials (RCTs) for the use of probiotic strains in the cases of chronic generalised periodontitis, concluding the beneficial effects of probiotic *Lactobacillus reuteri* in reversal to eubiosis state and/or improving clinical parameters [166]. The strains of lactic acid bacteria, *Lacticaseibacillus paracasei* 111 and *Lacticaseibacillus paracasei* 141, formulated into oral tablets were demonstrated to diminish the levels of periodontal pathogens by bacteriocin production and coaggregation activity in a clinical study [167].

In an interesting study by Terai et al. (2015), the effects of new probiotic candidates for potential oral health benefits were studied [168]. Out of 896 oral isolates derived from healthy subjects, 14 *Lactobacillus* and 36 *Streptococcus* strains showed key beneficial features such as no production of volatile sulphur compounds or water-insoluble glucan, exhibiting higher antibacterial activity against periodontal bacteria and higher adherence levels to oral epithelial cells [168]. Probiotics affect the oral cavity indirectly by modulating innate and adaptive immune function [169]. Lactic-acid-producing bacteria can interact with immunocompetent cells, such as macrophages and T-cells, causing alterations in cytokines production, leading to overall immunomodulation [169]. Considerable evidence related to the crucial role of the microbiome in cancer progression, targeting the microbiome could be used to enhance the efficacy of therapeutics, reduce their toxicity, and prevent cancer development [170]. The members of *Enterobacteriaceae*, *Fusobacterium*, *Haemophilus*, *Porphyromonas*, *Prevotella*, *Streptococcus* spp., and *Veillonella* have been found to be associated with pre-cancerous lesions and cancer in the oral cavity [24]. Numerous malignancies depend significantly on immune modulation. As probiotics play an important role in immune modulation, their efficacy has been thoroughly investigated in various cancer types. The probiotic products containing *Lactobacillus* and *Bifidobacterium* symbiotic associations in the form of pre-biotics are also a new concept to consider for the treatment of various oral diseases [171]. An earlier study also reported a similar concept of a symbiotic association of *Bifidobacterium longum* and *L. acidophilus*, displaying the inhibition of linoleic acid peroxidation as an antioxidant activity [172]. Thus, in relation to probiotic interventions as a prospected tool for the treatment or prevention of OC, the *Lactobacillus* strains have displayed various immunomodulatory, antioxidating, and apoptotic effects, in addition to DNA damage prevention and epigenetic mechanisms in literature [173].

Furthermore, *Lactobacilli* spp. secrete postbiotics (biogenics, metabolites, cell-free supernatants, and other waste products [174]) in the form of bacteriocin and reuterin (by *L. reuteri*) with a broad spectrum of antimicrobial activity, inhibiting the overgrowth of pathogenic as well as commensal bacteria [175,176]. In different studies, culture supernatants of *Lactobacillus rhamnosus* GG, *Lactococcus lactis* MG5125, and *Lactobacillus salivarius MG4265* were found to inhibit the biofilms of *Streptococcus mutans* indicative of the production of anticariogenic metabolites [176,177]. Various studies have explored the adjunctive therapy potential of probiotics expressing anticancer activity by secretion of anticancer agents, induction of apoptosis by means of reducing the COX2 (cyclooxygenase 2) expression (*L. salivarius* REN) as well as metabolite secretion (*Acidobacter syzygii*), and altering mRNA expressions (*L. plantarum*) [177,178,179,180]. In another context, nisin, a bacteriocin produced by the oral probiotic species has been determined to reverse the effects of the pathogens of the periodontal region, including *Porphyromonas gingivalis*, *Fusobacterium nucleatum*, and *Treponema denticola* that are actively involved in enhancing the aggressivity of OC via TLR/MyD88 mediated Integrin alpha5/FAK signalling [181].

Some recent advances in the identification of diverse oral microbiota have led to investigative studies of Oral Microbiota Transplant (OMT), where attempts have been made to transfer oral biofilms from a healthy donor to a patient with oral diseases such as caries or periodontitis in order to modify the oral environment and to maintain good oral health [182]. These studies have focused mainly on glycosylation-based bacterial cell adherence to oral cavity surfaces [183]. This concept is also supported by the expression of the arginine deiminase system (ADS) by a group of oral *streptococci* present in a substantial proportion of the oral microbiome [96]. *Streptococcus dentisani* or *Streptococcus* A12 have probiotic roles pertaining to preventing the growth of cariogenic species like *S. mutans* and change in acidic pH due to arginolytic activities, which critically contributes to pH homeostasis in oral biofilms [24,81,184]. Another study by Cheng et al. (2017) demonstrated the ability of *Lactobacillus rhamnosus* GG (LGG) to enhance the geniposide anticancer potential in human oral squamous carcinoma cells (HSC-3) [185]. The alpha diversity of the saliva microbiome increased significantly in the sample group that consumed the probiotic [155]. In our view, this should be investigated further to reverse the decreased alpha diversity observed in long-term BQ chewers [124].

In addition to the beneficial effects of probiotic microorganisms in the prevention or inhibition of OC, several studies reflect similar findings in the cases of cancer-therapy-induced oral mucositis. The potential of *Lactobacillus reuteri* to prevent oral mucositis by Nrf-2 signalling mediated decrement of oxidative stress, has been explored [186]. A meta-analysis of RCTs conducted in this regard revealed the significance of probiotics in the mitigation and prevention of mucositis as evidenced by low incidences of the disease in the probiotic groups [187]. Furthermore, according to a study utilising nanotherapeutics-based approach employing probiotic strains, a potential for better admissibility due to higher absorption and satisfactory targeted tumor activity in forms of oral medication, has been observed, which could be a better lead for utilising a microbiome approach with nanobiotechnological interventions for cancer patients [188]. In support of the following study, probiotics spores with chemotherapeutic drug activity can also be effectively utilised for their pharmacological efficiency to surpass the rigid molecular barriers, due to a similar colonising property in a rapid disintegrated hydrophobic protein form [189].

**Figure 5:**
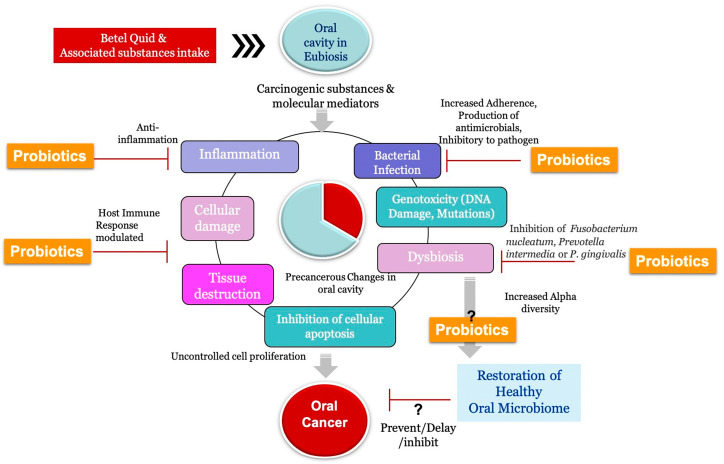
Prospecting Probiotic Approach for BQ-Driven OC and Local Dysbiosis Management.

Thus, probiotics seem to be a potential explorable approach to reverse the oral microbiota dysbiosis leading to OC due to BQ chewing and other such consumed substances. Detailed identification and comparisons of bacterial populations in the oral cavity under different conditions, such as among the healthy, pre-cancerous, and cancerous populations, may provide a lead for a promising probiotic consortium that could alter the oral microbial ecology in BQ chewers reversing or relieving the adverse impacts of BQ on oral dysbiosis and deteriorated oral health (Figure 5).

## 6. Conclusions and Future Prospects

Populations with high betel nut chewing rates, tobacco, and alcohol consumption [117,190] often exhibit a higher incidence rate of oral and pharyngeal cancers. OCs at early stages are generally asymptomatic, and patients typically seek medical attention only when the discomfort sets in. Thereby, the early benign stage of cancer is missed out, and only when there is growth, ulceration, pain, excessive salivation, loosening of teeth, and difficulty in swallowing do the patients seek medical help and advanced stages of OCs are diagnosed. Even after advances in cancer management, survival rates have not improved significantly. Therefore, innovative diagnostic approaches should be explored as a priority to identify patients at the pre-cancerous stage. This high-risk population of BQ chewers and people with habits of other substance abuse should be made aware of a regular oral mucosal check-up by dentists so that pre-cancerous lesions can be identified and treated. Fortunately, pre-cancerous lesions in the oral cavity can be detected through visual examination [191]. Numerous studies have pointed towards the role of oral bacteria in carcinogenesis [192,193,194] and confirmed the carcinogenic effects of *Fusobacterium nucleatum* and *Porphyromonas gingivalis* [28,115]; this could be good biomarker especially with vulnerabilities due to betel nut, alcohol abuse, smoking [31], and poor oral hygiene [195]. However, whether the imbalance in the oral microbiota results in further oncogenic progression is still a matter of debate [79]. Nevertheless, it can be safely proposed that the OSCC microenvironment “forces” the existent oral microbiome to ‘re-calibrate’ itself accordingly. This inference is further supported by the fact that the tumour or its adjacent buccal sites have bacteria with enhanced superoxide dismutase and lactoyl glutathione lyase activity, overcoming increased H_2_O_2_ and methylglyoxal in the tumour microenvironment [79,108]. Methylglyoxal can damage the biomolecules resulting in genotoxicity and disruption in cellular functions. Hence, the oral microbiome, being very responsive to changes in its environment, may be explored as a biomarker for the diagnosis of oral and oropharyngeal cancers [196]. These oral microbiome alterations will help in the fast diagnosis of cancer at its early stages if standardised protocols for sampling from different sites of the oral cavity are developed for accurate comparisons of healthy and cancer patient profiles with minimum error. The limitations of cultivation-based studies have been overcome following the approach of metagenomics. Detection using 16S rRNA gene-based molecular methods has certainly facilitated the discovery of previously undetected oral microbiome species and the association between the oral microbiota and OC. Metatranscriptomics and metaproteomics studies of such complex microbial communities as the oral microbiome can further advance this identification and comparison of the activities of microbes in different oral samples [197]. Yost et al. (2018) reported an increased expression of virulence genes in tumour and their adjacent sites, which could be related to cancer progression [79]. Similarly, another approach using metabolomics has been suggested for studying oral biofilms and OC [198,199]. Such studies will certainly enhance our understanding of oral microbiome dynamics and provide new directions on how dysbiosis arising in a different lifestyle and disease conditions can be successfully reversed. Moreover, with the success story of Faecal Microbiota Transplant (FMT) for the treatment of persistent *Clostridium difficile* infections, it is evident that the microbiome can be replenished and restored [200].

Thus, with regard to the reversal or restoration of oral dysbiosis to eubiosis state, there seem to be sufficient indicators that the probiotic approach can be helpful, as already achieved in the case of the gut microbiome. Further studies should be targeted towards the development of beneficial probiotic cocktails to restore oral cavity microbiota, for example, in BQ chewers to ameliorate its destructive effects through precise identification and detailed analysis of specific bacterial strains in oral microbiomes of healthy individuals. This probiotic-cocktail-based prophylactic treatment will require region/locale-specific customisation in terms of the composition of the specific probiotic bacterium. Moreover, a series of systematic epidemiological bacterial genome mapping studies, testing of specific bacterial species combinations, and clinical validation studies are warranted.

## Figures and Tables

**Table 1:** Studies connecting BQ chewing and other substances with OCs in Asian countries.

S. No.	Number of Subjects and Country of Study	Findings	References
1	42 OSCC patients, 45 OSF patients with areca chewing, 45 controls; China	Different abundance of bacterial taxa at different pathological stages of OSCC, areca chewing induces oral site-specific microbial alterations	[46]
2	20 subjects; Andhra Pradesh, India	The duration, frequency of betel nut, tobacco chewing, and overnight placement were statistically significant factors associated with potentially malignant lesions in oral cavity	[47]
3	2008 users Age: 20–80 years; Uttar Pradesh, India	High incidence of OPMDs in this population, associated with the smoking and BQ chewing habits	[48]
4	487 male Head and. Neck Cancer subjects (313 OC, 119 Oro- and hypopharyngeal cancers, and 55 Laryngeal cancers) and 617 controls; Taiwan	The highest Head and Neck Cancer risk associated with BQ was reported in buccal mucosa and gingiva of oral cavity	[49]
5	80 cases with OSF-associated OSCC and 80 controls with OSF but without evident OSCC; Hunan Province, Mainland China.	The use of BQ, cigarette, and alcohol identified as risk factors for malignant transformation of OSF in the synergistic effects between BQ chewing and cigarette or alcohol consumption were revealed	[50]
6	110 Subjects; Arunachal Pradesh, North East Region, India	Increased frequency of micro nuclei in buccal epithelium among smokers and alcohol consumers, BQ chewers and tobacco users compared to the control group.	[51]
7	81 cases of OC with 162 controls; Jakarta Indonesia	Smoking and BQ chewing are directly associated with OC risk. Chewing of at least one quid per day and betel leaf, AN, lime, and tobacco together caused a 5–6 times increased risk.	[37]
8	91 cases of Oesophageal Squamous Cell Carcinoma and 364 controls from three tertiary-care hospitals; Karachi, Pakistan	Several fold increase in the risk of oesophageal squamous-cell carcinoma, if the subjects smoked cigarettes and chewed BQ with tobacco	[52]
9	1522 Patients of aerodigestive tract OSCC; Taiwan	Groups with Alcohol, Betelnut and Cigarette and Alcohol, Tobacco-free BQ exhibited earlier diagnosis ages (10 years ahead) than non-users of these substances for OC	[53]
10	10,657 patients; Taiwan	Strong relationship between smoking, alcohol consumption, and BQ chewing for OC development	[54]
11	1029 subjects; Sri Lanka	Synergistic effect of chewing and alcohol consumption was reported as risk factors for oral potentially malignant disorders (OPMDs)	[55]
12	254 patients with oral Squamous Cell Carcinoma; Taiwan	BQ chewing and cigarette smoking patients are more likely to be diagnosed with oral cavity cancer at a younger age than those who have just one habit or none.	[56]
13	75 Habituates; North East Region, India	BQ chewers showed a significant increase in the binucleated cells in comparison to the non-chewers.	[57]
14	513 Patients of oesophagus Squamous cell carcinoma and 818 controls; Taiwan	Alcohol interacts with tobacco and BQ in a synergistic way in development of oesophageal cancer.	[58]
15	219 Patients with confirmed Oral Leukoplakia (OL) or OSF, and 876 randomly selected community controls; Taiwan	Tobacco smoking act synergistically along with BQ chewing to cause OL and OSF	[59]
16	Samples from 591 incident cases of oral cavity cancer and 582 hospital controls; Bangalore, Madras and Trivandrum, India	35% of OC in men was found attributable to the combination of smoking and alcohol consumption and 49% to pan-tobacco chewing.	[60]
17	79 subjects and 149 controls from hospitals; Pakistan	Subjects using quid without tobacco were 9.9 times more likely to develop OSCC as compared with non-users, those using with tobacco were at 8.4 times the controls, clearly showing association between use of paan without tobacco and OSCC	[61]
18	104 oral cancer patients compared with 194 controls; Taiwan	OC, 123-fold higher in patients who smoked, drank alcohol, and chewed BQ. Demonstrated synergistic effects of alcohol, tobacco smoke, and BQ in OC	[62]
19	143 OSCC; Natal, South Africa	AN habit with or without tobacco use was important in the development of OSCC	[63]

**Table 2:** Oral Microbiome alterations associated with OCs.

Bacteria	References	Clinically Useful Outcome	Summary of Key Findings
*Porphyromonas gingivalis*	[128]	May provide a diagnostic tool for OSCC	Increase in the abundance was noted in OC and leukoplakia.
*Bacteroidetes, Proteobacteria*	[129]	Suggested biomarkers for OSCC	Reduction in the abundance of phylum *Bacteroidetes* and increased detection of phylum *Proteobacteria* in OSCC tumour. Reported yeasts like *Rhodotorula, Geotrichum* and *Pneumocystis* to be linked with the tumour
	[35]		Reduction in the abundance of *Bacteroidetes* in tumour tissue.
*Neisseria*	[130]	Indicates a decrease in bacterial richness due to tobacco and alcohol abuse as a cause of oral health deterioration	Significant decrease in smokers
*Prevotella*			Significant reduction in smokers and drinkers.
	[131]	Poor oral hygiene correlated with OSCC	Associated with OSCC
*Capnocytophaga*	[130,132]	Indicates a decrease in bacterial richness due to tobacco and alcohol abuse as a cause of oral health deterioration	Significant reduction in smokers and drinkers.
*Actinobacteria*	[120,133]	Deviations in the abundance of oral microbiome related to OCC	Reduction in abundances in the tumour samples
*Fusobacterium nucleatum*; *Fusobacteria*	[116]	*F. nucleatum* as a prognostic marker	Linked with aggressive tumour behaviour through stimulation of chemokines
	[117,119,131,133,134,135]	Association of *F. nucleatum* and *Pseudomonas aeruginosa* with OSCC.	Increase in the abundance was noted in OC.
	[35]	Suggested biomarkers for OSCC	Reduction in the abundance in tumour tissue.
	[136]	Suggested biomarkers for OSCC including bacteria and their metabolites.	Higher abundance of pathogenic bacteria and lower abundance of commensals observed in tumour tissues compared to the non-tumour ones. Identified differential metabolic activities in the tumour tissue.
*Streptococcus anginosus*	[137]	*S. anginosus* of dental plaque linked to squamous cell carcinoma	*S. anginosus* isolated from the cancer tissue was identical to that from the dental plaque implying that dental plaque was the source site of pathogenic *S. anginosus*
Methanogenic archaea	[138]	Prevalence of methanogens in tobacco smokers and their transmission to non-smokers	Presence of *Methanobrevibacter oralis* and *M. smithii* in the oral fluid of tobacco smokers.

**Table 3:** Oral microbiome alterations concerning BQ and commonly associated abusive substances (tobacco and alcohol).

Bacterial rRNA Amplification & Sequencing	Subject Count & Mean Age (years)	Region	Results	References
V3–V4 region	N = 22	India	BQ chewers exhibit overall decreased bacterial diversity and richness	[23]
V3–V4 region	N = 43; Age—37.9 ± 6.19	Sri Lanka	Altered oral microflora in BQ chewers: increased abundance of periodontal pathogens (Actinomyces, Tannerella, and Prevotella), and decreased abundance of cariogenic pathogens (Streptococcus, Lautropia, and Actinobacillus)	[139]
V3–V5 region	N = 122; Age not provided	Guam	BQ chewers showing oral lesions higher levels of *Oribacterium*, *Actinomyces*, and *Streptococcus*	[124]
V1 region	N = 22; Age—58	Brazil	*Neisseria* abundance decreased in smokers and drinkers than controls. Smokers had a significant increase in *Prevotella* and *Capnocytophaga* and reductions in *Granulicatella*, *Staphylococcus*, *Peptostreptococcus*, and *Gemella*. Smokers/drinkers had lower levels of *Fusobacteria*	[130]
V4 region	N = 30; Age-62	USA	Reduction in *Firmicutes* and *Actinobacteria* while the increase in *Fusobacteria* in oral tumour samples of the smokers	[120]
V4 region	N = 252; Age—53	Taiwan	Higher abundance of *Cloacobacillus*, *Gemmiger*, *Oscillospora*, and *Roseburia* in the saliva of smokers and chewers than controls.	[19]
V4 region	N = 363; Age—58 (media)	USA	Higher abundance of *Dialister* in oral rinse samples of smokers than controls.	[119]
V3–V4 region	N = 55; Age not provided	Pakistan	Acidogenic and aciduric bacteria including *Veillonella, Streptococcus, Leptotrichia*, and *Serratia* showed increased abundance in BQ chewers.	[140]

## Data Availability

No new data were created or analysed in this study. Data sharing is not applicable to this article.
